# Paired comparison of tumor core and airway lumen (BALF) microbiomes in lung adenocarcinoma: deciphering specific *Bacillus* enrichment and immunomodulation

**DOI:** 10.3389/fcimb.2026.1768287

**Published:** 2026-07-06

**Authors:** Lingyu Yan, HaoShuai Yang, Hongxiang Feng, Fei Qi, Fanjia Kong, Qiduo Yu, Weijie Zhu, Jin Zhang, Chang Liu, Zhenrong Zhang

**Affiliations:** 1 Chinese Academy of Medical Sciences & Peking Union Medical College, Beijing, China; 2 Department of Thoracic Surgery, China-Japan Friendship Hospital, Beijing, China

**Keywords:** *Bacillus*, immune checkpoints, lung adenocarcinoma, spatial heterogeneity, tumor microenvironment

## Abstract

**Background:**

Lung adenocarcinoma (LUAD) is the leading cause of cancer mortality. While the lung microbiome influences tumorigenesis, the spatial heterogeneity between the airway reservoir (bronchoalveolar lavage fluid, BALF) and the actual intratumoral niche remains underexplored. Understanding this distinction is critical for identifying true tumor-resident drivers.

**Methods:**

Paired tumor tissue and BALF samples were collected from 77 LUAD patients. 16S rRNA gene sequencing targeting V3–V4 regions was performed to characterize microbial diversity. Host transcriptomics were integrated to explore host-microbe interactions. Bioinformatics analyses utilized UPARSE for OTU clustering, PICRUSt2 for functional prediction, and CIBERSORTx for immune cell deconvolution. Differential abundance was assessed using LEfSe and Random Forest algorithms to identify discriminatory biomarkers.

**Results:**

Comparative analysis revealed distinct ecological architectures, with tumor tissues exhibiting significantly reduced alpha-diversity compared to BALF. The genus *Bacillus* was identified as a key tumor-enriched biomarker (LDA > 2.0), distinct from airway colonizers. Clinically, elevated *Bacillus* abundance significantly correlated with tumor invasiveness. Immunologically, *Bacillus* load was inextricably linked to an immunosuppressive microenvironment, characterized by the upregulation of inhibitory checkpoints (VSIR, TIGIT, PD-L1) and a reduction in M1 macrophage infiltration. Functional analysis suggested *Bacillus* involvement in metabolic pathways facilitating tumor adaptation.

**Conclusion:**

This study delineates the spatial disparity between intratumoral and airway microbiomes, identifying *Bacillus* as a potential driver of malignant progression and immune evasion. Targeting intratumoral microbiota offers novel diagnostic and therapeutic avenues for lung cancer.

## 1 Introduction

As the leading cause of cancer-related mortality worldwide, lung cancer presents a formidable public health challenge, with lung adenocarcinoma (LUAD) constituting its most prevalent histological subtype (Siegel et al., 2023; Han et al., 2024). Consequently, elucidating the fundamental biological mechanisms driving LUAD tumorigenesis and progression is imperative for the development of novel diagnostic modalities and therapeutic interventions.

The microbiome has emerged as a key modulator of oncogenesis (Garrett, 2015), with pulmonary dysbiosis intrinsically linked to malignancy (Wypych et al., 2019). Previous studies have identified distinct microbial signatures in lung cancer, such as *Veillonella* enrichment in bronchoalveolar lavage fluid (BALF) (Lee et al., 2016) and specific intratumoral taxa correlated with relapse-free survival (Peters et al., 2019). These findings establish the lung microbiome as a functional component of the tumor microenvironment (TME).

However, most research relies on single-sampling modalities, precluding paired comparisons within the same individual. While BALF reflects the airway microbiota, the intratumoral microbiota represents the distinct ecosystem interacting directly with cancer cells (Nejman et al., 2020; Wong-Rolle et al., 2022).

To address this, the present study employed a paired design to investigate the spatial heterogeneity of the microbiome in LUAD, contrasting the airway reservoir (BALF) with the tumor core within the same patient cohort. Through the integration of 16S rRNA gene sequencing and host bulk RNA transcriptomics, this analysis extends beyond mere taxonomic profiling to dissect the functional crosstalk between intratumoral bacteria and host gene expression. Crucially, specific “tumor-resident” genera were prioritized to distinguish them from airway colonizers and to delineate their association with specific immune or oncogenic pathways. Furthermore, multiplexed fluorescence *in situ* hybridization (FISH) and immunofluorescence (IF) staining were implemented to provide definitive morphological and spatial evidence of microbial residency and localized host-microbe alignment within the intact tissue architecture.

Ultimately, this multi-omics approach aims to redefine the LUAD microecology, providing a robust scientific basis for discovering microbe-derived biomarkers and developing precision interventions targeting the tumor-associated microbiota (El Tekle and Garrett, 2023).

## 2 Material and methods

### 2.1 Study design and participant enrollment

This study was designed as an observational cohort study involving 77 patients diagnosed with lung adenocarcinoma (LUAD) who underwent surgical resection at the China-Japan Friendship Hospital. To ensure precise clinical phenotyping, pathological staging was determined in strict accordance with the 8th edition of the American Joint Committee on Cancer (AJCC) TNM staging system (Goldstraw et al., 2016). The entire study protocol underwent rigorous review and received approval from the Ethics Committee of the China-Japan Friendship Hospital (Approval No. ZRJY2021-TD04). Written informed consent was obtained from all participants prior to their inclusion in the study.

To minimize confounding factors that could influence the microbiome, strict inclusion and exclusion criteria were established. Inclusion criteria were: (1) patients aged between 18 and 80 years; (2) histologically confirmed diagnosis of LUAD following surgical resection; and (3) treatment-naive status with no prior history of anti-tumor therapy. Exclusion criteria comprised: (1) a history of other synchronous or metachronous malignancies; (2) prior exposure to neoadjuvant chemotherapy, radiotherapy, or surgical intervention for cancer; and (3) administration of antibiotics, probiotics (including any explicit dietary probiotic or prebiotic supplements), corticosteroids, or immunosuppressants within the one month preceding enrollment. This stringent criterion was enforced to rigidly minimize exogenous dietary or therapeutic microbial confounding factors. Detailed clinical and demographic characteristics of the cohort are summarized in **Supplementary Table 1**.

### 2.2 Sample collection and processing

Specimen acquisition was performed under aseptic conditions optimized for low-biomass lung microbiome studies.

Bronchoalveolar lavage fluid (BALF): BALF samples were collected using flexible bronchoscopy following local anesthesia with 2% lidocaine. To prevent upper respiratory tract contamination, the bronchoscope was advanced without suctioning until wedged in the target segment. A volume of 20–30 mL sterile 0.9% saline was instilled into the lung segment contralateral to the lesion and gently aspirated, yielding approximately 10 mL of fluid. Samples were immediately placed on ice and transported to the laboratory within 30 minutes. To enrich microbial biomass while depleting host contamination, a differential centrifugation protocol was employed: samples were first centrifuged at 300 × g for 10 minutes at 4 °C to pellet and remove host cells; the supernatant was then transferred to a new tube and centrifuged at ≥10,000 × g for 20 minutes at 4 °C to pellet microbial cells.

Tumor tissue: Tumor specimens were harvested aseptically immediately following surgical resection. Tissues were rinsed thoroughly with sterile saline to remove surface blood and debris, then flash-frozen in liquid nitrogen and stored at -80 °C until nucleic acid extraction.

Contamination control: Given the susceptibility of lung samples to environmental contamination, stringent quality control measures were implemented. Negative controls were processed alongside biological samples. DNA samples were required to meet rigorous quality standards (concentration > 1 ng/μL via Qubit; OD260/280 ratio of 1.8–2.0). Ultimately, 76 BALF samples and 69 tumor tissue samples passed quality filtering for sequencing.

### 2.3 DNA extraction and 16S rRNA sequencing

6S rRNA gene sequencing: Total microbial DNA was extracted from BALF pellets and tissue homogenates using the QIAamp DNA Microbiome Kit (Qiagen), strictly following the manufacturer’s protocol to maximize host DNA depletion. The V3–V4 hypervariable regions of the bacterial 16S rRNA gene were amplified using universal primers 341F (5’-ACTCCTACGGGAGGCAGCA-3’) and 806R (5’-GGACTACHVGGGTWTCTAAT-3’) (Klindworth et al., 2012). The PCR reaction system (20 µL) contained 4 µL 5× FastPfu Buffer, 2 µL 2.5 mM dNTPs, 0.8 µL each primer (5 µM), 0.4 µL FastPfu Polymerase, and 10 ng template DNA. Amplification was performed on an ABI GeneAmp^®^ 9700 system: 95 °C for 3 min; 27 cycles of 95 °C for 30 s, 55 °C for 30 s, and 72 °C for 45 s; followed by 72 °C for 10 min. Amplicons were purified using the AxyPrep DNA Gel Extraction Kit (Axygen) and quantified using the Qubit™ dsDNA HS Assay Kit. Equimolar pools were sequenced on an Illumina NovaSeq PE250 platform (Shanghai Majorbio Bio-pharm Technology Co., Ltd.).

Transcriptomic sequencing: To investigate host gene expression, total RNA was extracted from tumor tissues of 53 patients, representing the subset with paired microbiome data, using Trizol reagent (Invitrogen). RNA integrity was verified using an Agilent 2100 Bioanalyzer. mRNA was enriched using Oligo(dT) beads, fragmented, and converted to cDNA. Libraries were constructed using the NEBNext Ultra RNA Library Prep Kit (NEB, USA) involving end repair, A-tailing, adaptor ligation, and PCR amplification. Sequencing was performed on an Illumina NovaSeq X Plus platform (Hangzhou Astrocyte Technology Co,.Ltd) to generate paired-end reads.

### 2.4 Bioinformatics and microbiome analysis

Sequence processing and taxonomy: Raw 16S rRNA gene sequencing data were demultiplexed and quality-filtered using fastp (v0.23.4) to remove adapters and low-quality bases. Paired-end reads were merged using FLASH (v1.2.11). Operational Taxonomic Units (OTUs) were clustered at a 97% similarity threshold using UPARSE (v7.0) (Edgar, 2013), with chimeric sequences identified and removed. Taxonomic assignment was performed using the RDP Classifier (v2.13) against the Silva 16S rRNA database (Release 138) with a confidence threshold of 0.7. Strict decontamination procedures were applied: OTUs annotated as mitochondria, chloroplasts, or non-bacterial domains were filtered out, and potential contaminant sequences identified in negative controls were bioinformatically subtracted. Specifically, negative controls, including sterile saline washes and empty extraction kit reagents were sequenced alongside biological specimens. *Bacillus* sequences were either entirely absent or detected at negligible baseline levels (relative abundance < 0.01%) in these negative controls. Potential contaminant operational taxonomic units (OTUs) identified in the controls were bioinformatically subtracted from the final dataset using the microDecon package to ensure taxonomic authenticity. To normalize for sequencing depth, OTU abundance tables were rarefied to the minimum sequencing depth across samples.

Multiplex fluorescence *in situ* hybridization combined with immunofluorescence: To validate the physical presence and visualize the microenvironmental localization of the *Bacillus* genus within lung adenocarcinoma (LUAD) tissues, multiplex FISH-IF staining was performed on formalin-fixed paraffin-embedded (FFPE) continuous tissue sections (10–20 μm thickness). A two-tier staining framework was implemented: a three-color cellular compartment panel was applied to six sections, and a parallel four-color immune checkpoint panel was applied to another six sections.

For bacterial detection, a specific *Bacillus* genus-targeted 16S rRNA oligonucleotide probe LGC353b (5’-GCG GAA GAT TCC CTA CTG C-3’) and a universal bacterial probe EUB338 (5’-GCT GCC TCC CGT AGG AGT-3’) were synthesized and fluorophore-labeled at the 5’ end. Sections underwent deparaffinization and hybridization in a buffer containing 20% formamide with the respective probes at 46 °C. Following hybridization, the three-color panel slides were incubated with primary antibodies against Cytokeratin (CK) and CD45, while the four-color panel slides were incubated with antibodies against CK, CTLA-4, and PD-1 overnight at 4 °C. Slides were then incubated with corresponding fluorophore-conjugated secondary antibodies, and nuclei were counterstained with DAPI.

High-resolution tile scans were acquired using a fluorescence scanner. Quantitative analysis of *Bacillus* puncta density (n = 32 standardized fields per tissue region), metric-based distance-transform spatial proximity (n = 16 core fields), and the mean fluorescence intensity (MFI) of immune checkpoints (n = 8 niches per group) was executed using ImageJ (FIJI) software.

Microbiome profiling and biomarker discovery: Alpha diversity was assessed using Chao1 and Shannon indices. Beta diversity was visualized via Principal Coordinates Analysis (PCoA) based on Bray-Curtis dissimilarity matrices. Differential abundance analysis was performed using LEfSe (Linear Discriminant Analysis Effect Size) with an LDA score threshold of >2.0 to identify taxa driving differences between groups. A Random Forest classification model was established to validate the discriminatory power of these markers. Microbial co-occurrence networks were constructed based on Spearman correlations (|ρ| > 0.6, P < 0.05) and visualized using Gephi to identify keystone taxa. Functional prediction was conducted using PICRUSt2 to map OTUs to COG and KEGG pathways.

### 2.5 Functional prediction and immune correlation

Functional profiling: Phylogenetic Investigation of Communities by Reconstruction of Unobserved States 2 (PICRUSt2) (Douglas et al., 2020) was utilized to predict the functional potential of the microbiome. OTUs were mapped to COG and KEGG pathways to identify metabolic functions significantly enriched in different groups.

Multi-omics integration: To elucidate host-microbe interactions, microbiome data were integrated with transcriptomic profiles. The CIBERSORTx algorithm (Newman et al., 2019) was employed to deconvolute the relative proportions of 22 infiltrating immune cell types in the tumor microenvironment. Spearman correlation analyses were conducted to link the abundance of core microbial genera with: (1) immune cell infiltration levels; and (2) the expression of canonical immune checkpoint genes. Furthermore, a Protein-Protein Interaction (PPI) network was constructed by mapping host genes significantly correlated with core microbes to the STRING database (v11.5, interaction score > 0.4). The network was visualized in Cytoscape, and functional enrichment (GO and KEGG) was performed on key network nodes to identify biological pathways modulated by the microbiome.

### 2.6 Statistical analysis

All statistical computations were executed using R software (v4.2.1) and the MicrobiomeAnalyst platform. Continuous variables were expressed as mean ± standard deviation (SD) or median (interquartile range, IQR). Group comparisons were performed using Student’s t-test or Wilcoxon rank-sum test for two groups, and ANOVA or Kruskal-Wallis test for multiple groups, depending on data normality. Correlation strength was assessed via Spearman’s rank correlation coefficients. All statistical tests were two-sided, and a P-value < 0.05 was considered statistically significant. To maintain high taxonomic accuracy, the International Code of Nomenclature of Prokaryotes (ICNP) for all bacterial naming conventions are followed (Oren and Garrity, 2021).

## 3 Results

### 3.1 Overview of microbial communities in tumor tissue and BALF

**Figure 1:**
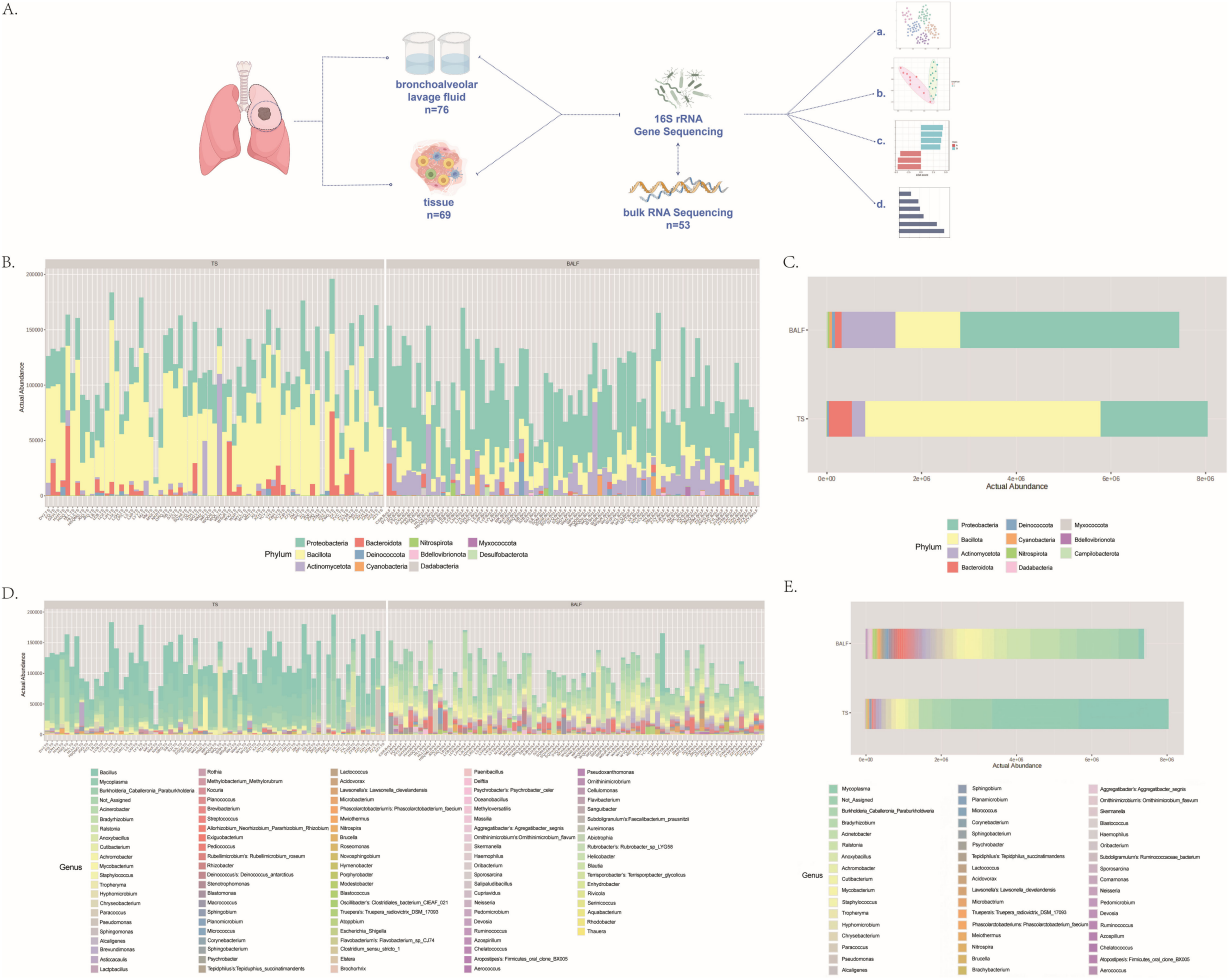
Study design and compositional landscape of the lung adenocarcinoma microbiome. **(A)**Schematic workflow. Overview of the paired study design, sample collection (Tumor Tissue vs. BALF), and the sequencing analysis pipeline. **(B, C)** Phylum-level composition. **(B)** Stacked bar charts and **(C)** pie charts illustrating the relative abundance of dominant bacterial phyla, highlighting the proportional shifts between the tumor and airway environments. **(D, E)** Genus-level profiling. **(D)** Taxonomic distribution and **(E)** comparative analysis of the most abundant bacterial genera, revealing distinct ecological signatures in the intratumoral niche compared to the airway lumen.

16S rRNA sequencing was performed on 76 bronchoalveolar lavage fluid (BALF) samples and 69 lung adenocarcinoma intratumoral tissue (TS) samples (**Figure 1A**). The analysis revealed significant disparities in the taxonomic composition of microbial communities between the two anatomical niches.

At the phylum level, although *Pseudomonadota, Bacillota, Actinomycetota*, and *Bacteroidota* constituted the predominant phyla in both groups (**Figure 1B**), their relative abundances exhibited marked variation. Statistical analysis confirmed that *Bacillota* and *Bacteroidota* were significantly enriched in the intratumoral tissues, whereas *Pseudomonadota* and *Actinomycetota* were significantly more abundant in the BALF (**Figure 1C**).

These compositional differences were further accentuated at the genus level. The overall genus profile (**Figure 1D**) demonstrated that the two groups were dominated by distinct taxa. The microbial community within the TS samples appeared relatively conserved and was primarily dominated by a limited number of genera, with a significantly elevated relative abundance of *Bacillus* and *Mycoplasma*. In sharp contrast, the BALF samples exhibited a more diverse microbial ecosystem. The dominant microbiota in BALF mainly comprised *Planococcus, Mycobacterium, Streptococcus, Haemophilus, Neisseria, Prevotella, and Porphyromonas*. The relative abundances of these genera were significantly higher in the BALF group compared to the TS group, collectively defining the unique microbial signature of the airway reservoir.

### 3.2 Differences in microbial diversity and community structure

**Figure 2:**
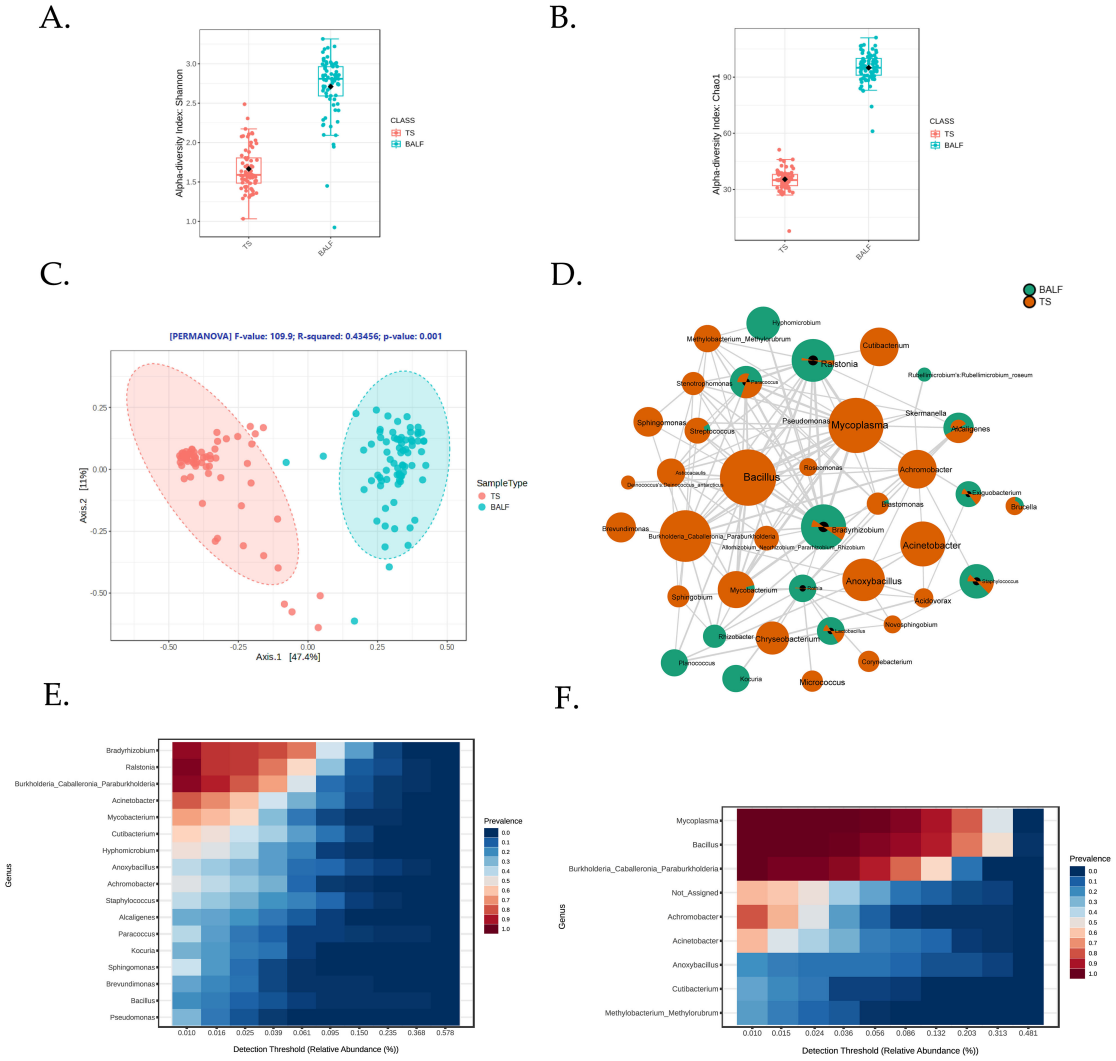
Ecological diversity and structural topology of the microbial communities. **(A, B)** Alpha-diversity analysis. Boxplots representing the **(A)** Shannon and **(B)** Chao1 indices. Statistical comparisons indicate significant differences in microbial richness and evenness between the groups (P < 0.01). **(C)** Beta-diversity analysis. Principal Coordinates Analysis (PCoA) based on Bray-Curtis dissimilarity, visualizing the distinct clustering patterns and structural heterogeneity of the microbiomes (PERMANOVA, P = 0.001). **(D)** Co-occurrence network analysis. Network diagrams displaying the interaction patterns among dominant taxa, highlighting the divergent topological features and ecological connectivity within the tumor microenvironment versus the airway. **(E, F)** Core microbiome analysis. Venn diagrams illustrating the shared and unique core genera (present in >20% of samples) in the BALF **(E)** and TS **(F)** groups. Overlapping regions indicate compositional connectivity between the airway and tumor, while non-overlapping areas highlight niche-specific colonization.

Alpha diversity analysis indicated that the BALF microbiome possessed greater community richness and evenness compared to the TS microbiome, as evidenced by significantly higher Shannon (p < 0.01) and Chao1 indices (p < 0.01) (**Figures 2A, B**). Regarding Beta diversity, Principal Coordinate Analysis (PCoA) demonstrated distinct clustering in two-dimensional space, highlighting a clear separation between the TS and BALF cohorts. Network analysis further elucidated the distinct ecological structures: the BALF network was anchored by dominant species such as *Bradyrhizobium*, *Acinetobacter*, *Paraburkholderia*, *Rothia* and *Ralstonia*. Conversely, the TS network was primarily orchestrated by genera including *Mycoplasma, Bacillus, Achromobacter, Anoxybacillus and Streptococcus*, which occupied central positions within the co-occurrence network (**Figure 2D**).

Core microbiome analysis provided additional evidence of this ecological heterogeneity. At the genus level, the core microbiota of the BALF group predominantly included *Bradyrhizobium, Ralstonia, Burkholderia-Caballeronia-Paraburkholderia, Acinetobacter, Mycobacterium, Cutibacterium*, and *Hyphomicrobium* (**Figure 2E**). In contrast, the core microbiota of the TS group was characterized by *Mycoplasma, Bacillus, Burkholderia-Caballeronia-Paraburkholderia, Achromobacter*, and *Acinetobacter* (**Figure 2F**). The prevalence of these dominant taxa differed between the two groups, as detailed in **Supplementary Table 2**. Notably, while genera such as *Acinetobacter* and *Burkholderia-Caballeronia-Paraburkholderia* were share between the two compartments—indicating a compositional overlap—the presence of unique core taxa in each group highlights niche specificity. Consequently, these core compositions demonstrate that the intratumoral tissue and the airway fluid represent two relatively independent microbial microenvironments, despite sharing partial ecological connectivity.

### 3.3 Screening and identification of key differential genera

**Figure 3:**
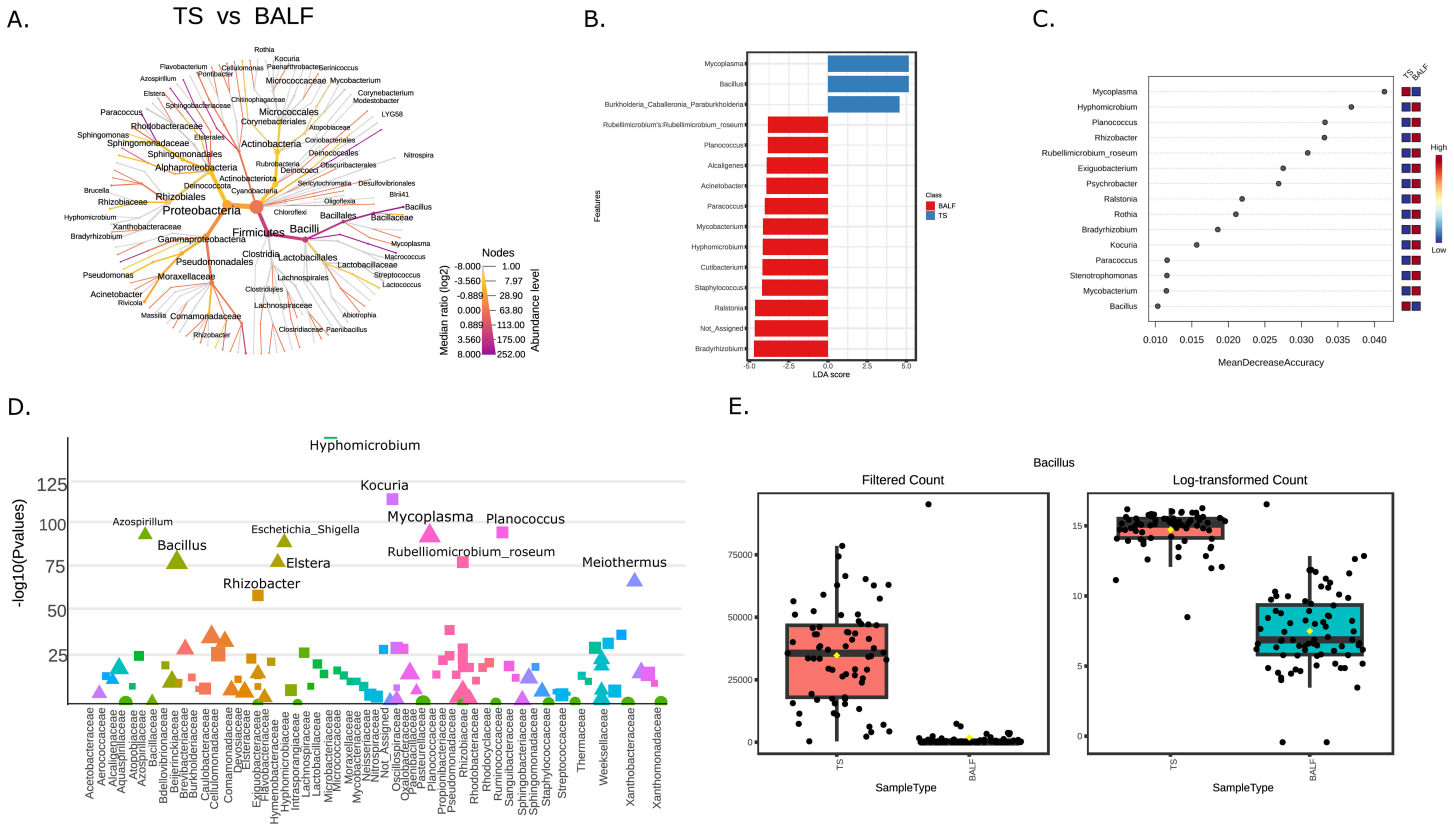
Identification and validation of tumor-enriched bacterial biomarkers. **(A)** Phylogenetic visualization. Heat tree analysis depicting the taxonomic discrepancies and lineage-specific differences between the two groups. **(B)** Differential abundance analysis (LEfSe). Histogram of LDA scores identifying specific bacterial taxa that statistically distinguish the tumor tissues from BALF (LDA score > 2.0, P < 0.05). **(C)** Feature importance ranking. Random Forest classification plot displaying the top discriminatory bacterial genera ranked by their Mean Decrease Gini scores, confirming the predictive value of the identified candidates. **(D)** Detailed abundance profile. Comparative visualization of the specific candidate genus across all samples. The distribution pattern and directional indicators signify a statistically significant enrichment in the tumor microenvironment compared to the airway (P < 0.001). **(E)** Biomarker selection strategy. A schematic summary of the multi-step screening pipeline—integrating LEfSe, Random Forest, and univariate statistics—used to prioritize *Bacillus* as the potential driver for downstream investigation.

To pinpoint the specific taxa driving the divergence between the two groups, a multi-step analytical approach was utilized. First, Heat tree analysis visually illustrated the abundance differences across taxonomic levels (**Figure 3A**). The *Bacillota* branch appeared robustly purple, with the *Bacilli* class and *Bacillus* genus nodes being particularly prominent, confirming their dominance in the TS group. Conversely, the *Pseudomonadota* branch was largely orange, with the *Acinetobacter* node (within *Gammapseudomonadota*) appearing significant.

Subsequently, LEfSe analysis identified 3 genera significantly enriched in the TS group and 12 in the BALF group (LDA > 2.0, p < 0.05). Notably, *Mycoplasma, Bacillus*, *and* *Burkholderia-Caballeronia-Paraburkholderia* were enriched in the TS group, whereas *Bradyrhizobium, Ralstonia, Staphylococcus, Cutibacterium*, and *Hyphomicrobium* scored higher in the BALF group (**Figure 3B**).The detailed statistical metrics, including P-values, FDR, and LDA scores for these biomarkers, are provided in **Supplementary Table 3**. To further isolate the most discriminatory features, a Random Forest model was constructed. Among the top 15 important genera identified, *Mycoplasma* and *Bacillus* were the primary contributors to the classification of the TS group. For the BALF group, key contributors included *Hyphomicrobium*, *Planococcus, Rhizobacter*, and *Rubellimicrobium-roseum*. Univariate analysis further corroborated that *Bacillus* abundance was significantly higher in the TS group compared to the BALF group (p < 0.001) (**Figure 3D**).

Collectively, these analyses converged on *Bacillus* as the core differential genus responsible for the observed ecological shift. Consequently, *Bacillus* was selected as the focal target for subsequent in-depth investigation. Comparative analysis confirmed a significant differential expression of *Bacillus* between TS and BALF samples (**Figure 3E**).

#### 3.3.1 Functional profile analysis of *Bacillus*

**Figure 4:**
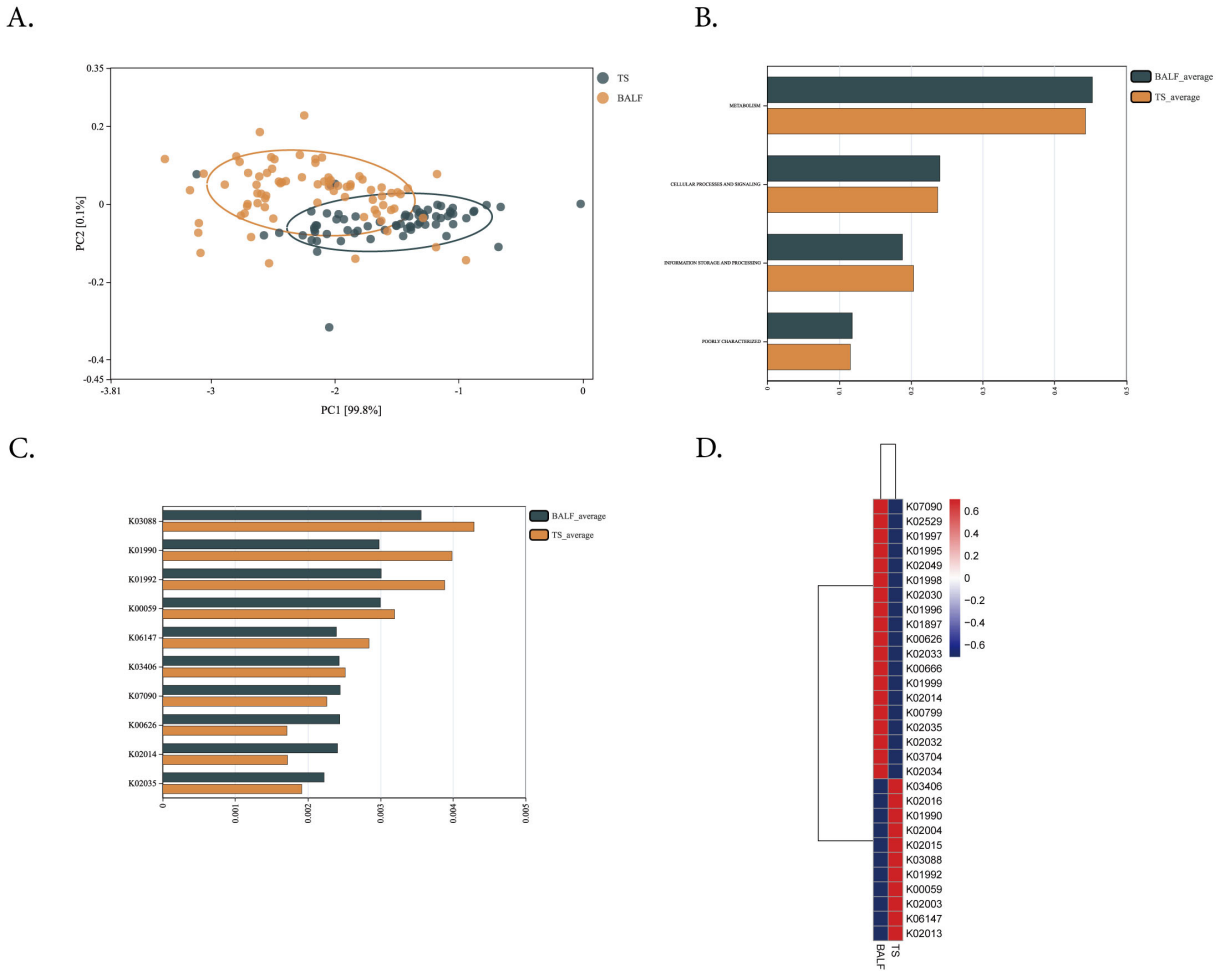
Predicted functional profiling and metabolic potential. **(A)** Functional Beta-diversity. Principal Coordinates Analysis (PCoA) based on the Bray-Curtis dissimilarity of predicted functional profiles (COG/KEGG), visualizing the distinct metabolic potential of the tumor-resident microbiome compared to the airway microbiota. **(B)** Functional enrichment analysis. Bar charts illustrating the specific functional categories that are significantly overrepresented in the tumor group. **(C)** KEGG pathway analysis. Bar charts showing the significantly enriched metabolic pathways, suggesting the active metabolic involvement. **(D)** Metabolic inference. Conceptual diagram or mapping suggesting the potential involvement of the identified microbiota in nutrient metabolism and microenvironmental cross-talk.

To explore the functional implications of these microbial variations, functional profiles were predicted based on the COG and KEGG databases. Principal Component Analysis (PCA) based on COG functional annotation revealed that while there was some overlap, the functional landscapes of the TS and BALF groups were largely distinct. A detailed comparison of COG categories (**Figure 4B**; detailed statistics in **Supplementary Table 4**) indicated that the highly enriched pathways were related to Metabolism, Cellular Processes and Signaling, Information Storage and Processing.

Furthermore, KEGG pathway enrichment analysis highlighted the top ten enriched pathways for each group (**Figure 4C**). The specific P-values and abundance data for these pathways are listed in **Supplementary Table 5**. Heatmap visualization of these pathways (**Figure 4D**) demonstrated that pathways K03088, K01990, K01992, and K06147 were highly enriched in the TS group, whereas pathways K00626, K02014, and K02034 were significantly enriched in BALF. Collectively, these functional predictions reveal that the intratumoral microbiota possesses specific metabolic capabilities distinct from those in the airway, particularly in pathways related to metabolism and cellular processes.

### 3.4 Clinical characteristics and immune microenvironment association of *Bacillus*

**Figure 5:**
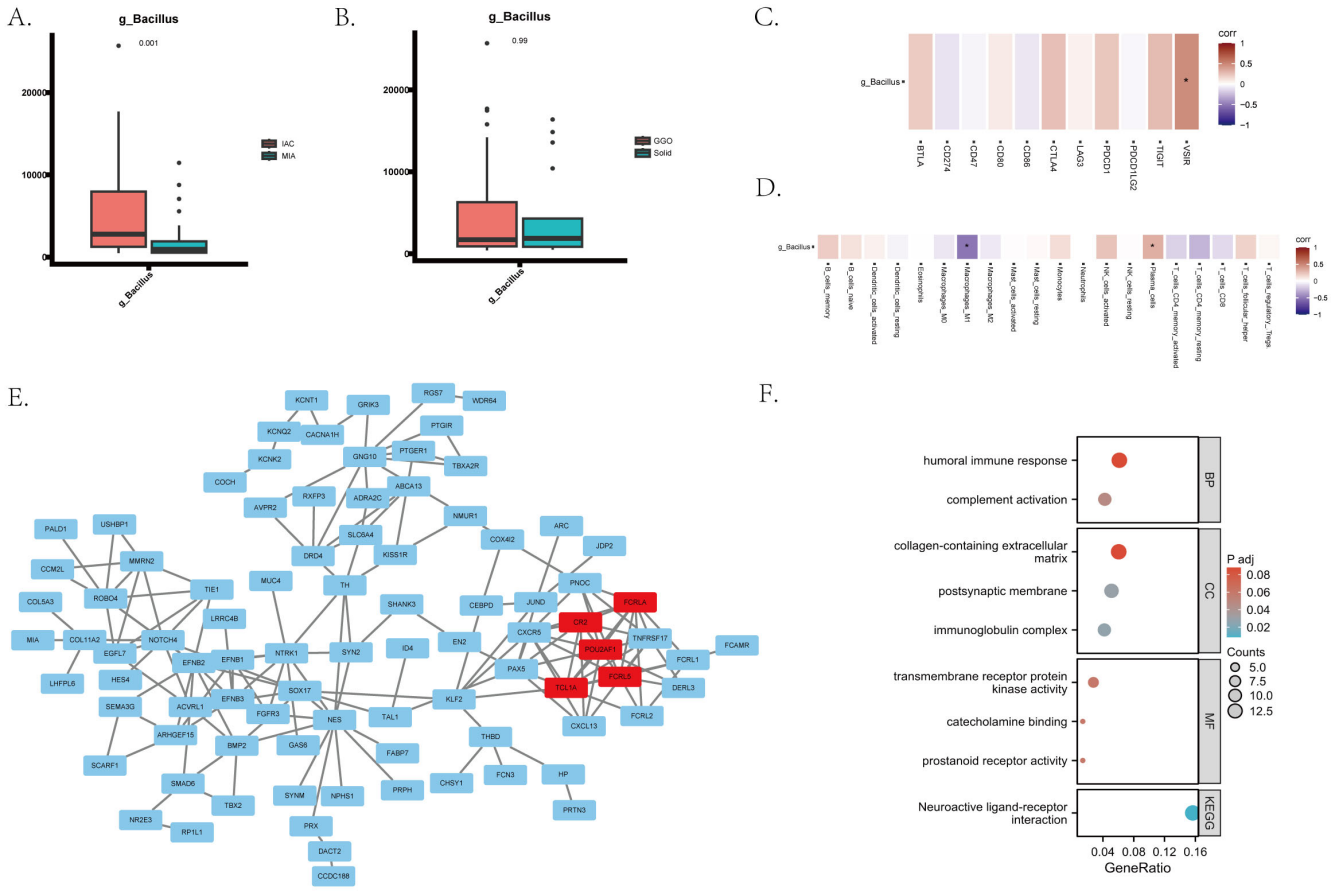
Clinical relevance and immunological landscape associated with the intratumoral microbiome. **(A, B)** Clinicopathological associations. Comparison of specific microbial abundance across different clinical subgroups, including **(A)** tumor invasiveness (IAC vs. MIA) and **(B)** radiological features (Solid vs. GGO) (P < 0.05). **(D)** Immune checkpoint correlation. Heatmap displaying Spearman correlations between microbial load and the expression of key immune checkpoint genes (e.g., PD-L1, CTLA-4). **(E)** Immune cell infiltration. Analysis of immune cell proportions in relation to microbial abundance, highlighting the shift in the immune microenvironment. **(E, F)** Host-microbe interaction. **(E)** Protein-Protein Interaction (PPI) network and **(F)** functional enrichment analysis (GO/KEGG) of host genes correlated with the key microbial marker, suggesting potential signaling pathways modulated by the microbiome.

The association among *Bacillus* abundance, LUAD clinicopathological features, and the immune microenvironment was subsequently evaluated. It was observed that *Bacillus* abundance was significantly elevated in invasive adenocarcinoma (IAC) compared to minimally invasive adenocarcinoma (MIA) (p = 0.001) (**Figure 5A**). However, no significant difference in distribution was found between solid nodules and ground-glass nodules (p = 0.99) (**Figure 5B**), suggesting that *Bacillus* may be specifically linked to the invasive progression of LUAD rather than radiographic density alone.

Immune association analysis revealed that *Bacillus* abundance was closely correlated with the expression of key immune checkpoint molecules. A highly significant positive correlation was observed with VSIR expression, alongside positive correlations with TIGIT, PDCD1, CTLA4, and BTLA (**Figure 5C**). Additionally, deconvolution analysis of immune cell infiltration via CIBERSORT further suggested the immunomodulatory role of *Bacillus*. The abundance of *Bacillus* exhibited a statistically significant positive correlation with the infiltration of plasma cells, memory B cells, activated NK cells, and follicular helper T cells (Tfh). Conversely, a significant negative correlation was identified with M1 macrophage infiltration, as well as with the levels of resting CD4 memory T cells, activated CD4+ memory T cells, and CD8+ T cells (**Figure 5D**).

Finally, a Protein-Protein Interaction (PPI) network was constructed incorporating all genes correlated with *Bacillus* expression, identifying POU2AF1, CR2, FCRL5, FCRLA, and TCL1A as hub genes. To further elucidate the mechanism by which *Bacillus* influences tumor development, GO and KEGG pathway enrichment analyses were performed on these correlated genes (**Figure 5F**). The results demonstrated significant enrichment in pathways related to the humoral immune response, collagen-containing extracellular matrix, and transmembrane receptor protein kinase activity.

#### 3.4.1 *In situ* validation of intratumoral *Bacillus* and its spatial proximity to epithelial and immunosuppressive compartments

To substantiate our bioinformatic predictions regarding the clinical and immunological relevance of the intratumoral microbiota, we performed multiplexed *in situ* imaging via joint FISH-IF on clinical LUAD sections. Universal bacterial mapping (EUB338+) validated the authentic intra-tissue colonization of bacteria within the tumor core. Crucially, utilizing the genus-specific LGC353b probe, we successfully visualized distinct, punctate *Bacillus* signals structurally embedded within the cellular architecture of LUAD.

**Figure 6:**
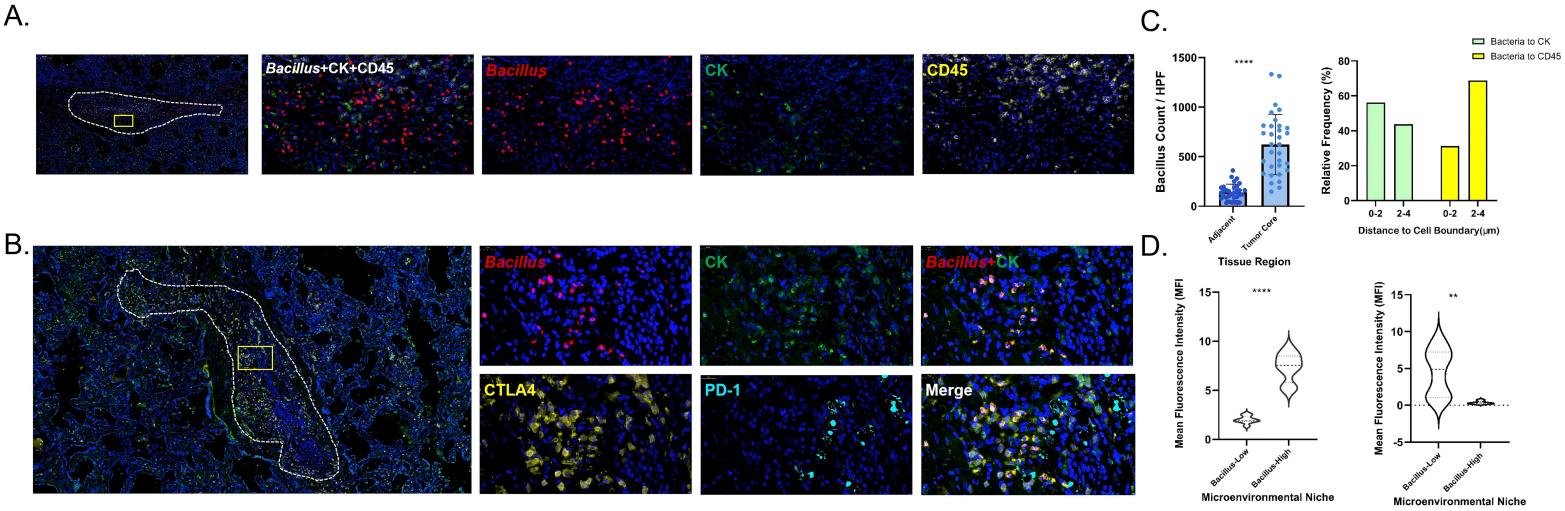
*In situ* multiplex validation of intratumoral *Bacillus* and its spatial correlation with cellular compartments and divergent inhibitory checkpoints. **(A)** Representative multiplex fluorescence *in situ* hybridization and immunofluorescence (FISH-IF) images from the three-color cellular panel. Left panel illustrates a low-power global overview of human LUAD tissue architecture with an annotated region of interest (ROI). Right panels illustrate high-power split-channel views demonstrating the topological co-localization of *Bacillus* puncta (LGC353b probe, red) with CK+ tumor epithelial cells (green) and CD45+ total infiltrating host leukocytes (yellow). Nuclei are counterstained with DAPI (blue). **(B)** Representative multiplex FISH-IF images from the four-color checkpoint panel. Left panel illustrates a low-power global view with a highlighted ROI. Right panels illustrate high-power split-channel views displaying the spatial distribution of *Bacillus* puncta (red) and CK+ tumor cells (green) with localized profiles of host CTLA-4 (yellow) and PD-1 (cyan) expressions. **(C)** Tissue-level spatial quantifications derived from the three-color cellular cohort. Left plot depicts the comparative analysis of absolute *Bacillus* puncta density between adjacent non-tumor tissues and LUAD tumor cores (n = 32 standardized fields per group, Unpaired t-test, ****P < 0.0001). Right plot depicts the metric-based distance-transform frequency distribution histogram, showcasing the relative frequencies (%) of the minimal physical distance from individual bacterial cells to the nearest CK+ tumor cell boundaries (green bars) or CD45+ leukocyte boundaries (yellow bars) stratified into 2 μm intervals (n = 16 core fields). **(D)** *In situ* fluorometric assessment of host immune checkpoints derived from the four-color panel (n = 8 microenvironmental niches per group, Welch’s corrected unpaired t-test). Violin plots illustrate the significant upregulation of CTLA-4 mean fluorescence intensity (MFI) within *Bacillus*-high niches (left plot, yellow, ****P < 0.0001$) and the inverse enrichment of PD-1 MFI within *Bacillus*-low niches (right plot, cyan, P = 0.0075). Horizontal dashed lines within violins represent the median and interquartile ranges.

Quantitative biomass characterization demonstrated that *Bacillus* absolute abundance was significantly enriched within the tumor core microenvironment compared to adjacent non-tumor tissue areas (623.5 ± 55.76 vs. 140.9 ± 55.76 counts/HPF, P < 0.0001; **Figure 6C**). Spatial proximity transform modeling further revealed that *Bacillus* was not randomly distributed; instead, the quantified bacterial population resided within a 4 μm physical radius from host cells. Specifically, *Bacillus* clusters exhibited close proximity to CK+ tumor epithelial cells, with a mean physical distance of 1.768 ± 1.211 μm, where 55.6% of the population nested within the immediate 0–2 μm intracellular/border zone. Concurrently, *Bacillus* was extensively intercalated within dense aggregates of CD45+ leukocytes, exhibiting a mean absolute distance of 2.260 ± 0.695 μm, with 68.9% clustering within the 2–4 μm peri-leukocytic niche (**Figure 6C**).

Furthermore, in alignment with our transcriptomic correlation framework, quantitative MFI evaluation demonstrated a pronounced yet divergent enrichment of inhibitory immune checkpoints within different microenvironmental niches. The expression of host CTLA-4 MFI was profoundly upregulated on leukocytes located in immediate proximity to high-load *Bacillus* niches compared to *Bacillus*-low regions (7.351± 0.5412 vs. 2.008 ± 0.5412, P < 0.0001; **Figure 6D**). Intriguingly, an inverse spatial topology was observed for the PD-1 checkpoint, where the MFI of localized PD-1 was significantly enriched within *Bacillus*-low peripheral niches relative to microbe-dense cores (4.489 ± 1.117 vs. 0.3565 ± 1.117, P = 0.0075; **Figure 6D**). Taken together, these histological observations provide robust morphological proof that tumor-resident *Bacillus* physically aligns with the host tumor-immune interface, establishing a highly compartmentalized, immunosuppressive microenvironmental niche in LUAD.

## 4 Discussion

This study systematically compared TS and paired BALF, revealing significant heterogeneity in microbial diversity and community structure. The core finding is that *Bacillus* is significantly enriched in TS compared to BALF, marking a distinct intratumoral ecosystem. Furthermore, the abundance of this genus correlates closely with tumor invasiveness and an immunosuppressive microenvironment.

While the lung was historically viewed as sterile, emerging evidence confirms the presence of an unique microbiota (Sepich-Poore et al., 2021, Nejman et al., 2020). However, the spatial distinction between the solid tumor and the fluid-based airway environment remains poorly characterized. In this study, we observed significantly lower alpha-diversity in TS compared to BALF, indicating a marked diversity drop within the tumor core. This finding mirrors the reduced diversity seen in non-malignant lung tissues and the gut of LUAD patients (Guo et al., 2023; Yang et al., 2024), likely reflecting selective pressures exerted by the tumor microenvironment (TME). Factors such as hypoxia, acidosis, and nutrient competition presumably select for taxa capable of surviving in these hostile conditions. This ecological filtering explains our observation of the core microbiome: although anatomical continuity facilitates partial microbial exchange, the tumor core maintains a distinct ‘privileged’ niche that restricts colonization to specific, adapted genera. Furthermore, the distinct clustering of TS and BALF samples highlights high environmental specificity, mirroring studies showing that anatomical location—such as oral versus lung—dictates microbial composition (Vogtmann et al., 2022; Yifan Sun et al., 2023). Notably, even when overall diversity remains comparable, the taxonomic composition of malignant lesions differs clearly from benign ones (Yang et al., 2025), underscoring the decisive role of the local microenvironment in sculpting specific ecological niches.

Beyond taxonomic differences, we observed significant functional divergence. Functional prediction analysis highlighted an enrichment of pathways related to metabolism, cellular processes, and information processing within the tumor microbiome, suggesting that resident microbes may actively modulate the TME via metabolic reprogramming. Consistent with this, tumor-resident *Staphylococcus* has been shown to secrete lactate to upregulate MCT1 expression and activate pseudo-hypoxic pathways, thereby promoting metastasis (Yu et al., 2025). Similarly, the gut microbiota and its derived metabolites are known to influence responses to chemotherapy, radiotherapy, and immunotherapy in non-small cell lung cancer (NSCLC). Collectively, these findings imply that the microbiome is not a passive bystander but an active participant in tumor progression, exerting influence through multifaceted interactions spanning from metabolic regulation to therapeutic response.

To pinpoint the specific taxa driving these functional perturbations, we employed a rigorous strategy integrating LEfSe, Random Forest (RF), and logistic regression algorithms. This approach robustly identified *Bacillus* as a key differential genus within the TS group. This finding aligns with reports of *Bacillus* enrichment across various lung cancer subtypes, particularly NSCLC and adenocarcinoma (Yan et al., 2025). Broader evidence from The Cancer Genome Atlas (TCGA) further links variations in bacterial community structure to clinical stages (Su et al., 2024), while longitudinal studies tracking the evolution from adenocarcinoma *in situ* (AIS) to invasive adenocarcinoma (IAC) have documented dynamic microbial shifts associated with invasive progression (Yang et al., 2024). Consistent with these precedents, our study observed a positive correlation between *Bacillus* abundance and clinical features of invasiveness. This is further corroborated by recent research showing that *Bacillus* levels rise significantly during the transition from benign lesions to the invasive phase (Du et al., 2025). Collectively, these converging lines of evidence suggest that *Bacillus* is not merely a passenger, but a potential driver or facilitator of malignant progression in lung adenocarcinoma.

Beyond metabolic interactions, a pivotal finding of this study is the intricate association between *Bacillus* and the tumor immune microenvironment (TIME). High *Bacillus* abundance was significantly correlated with the upregulation of inhibitory immune checkpoint molecules, particularly VSIR (VISTA), which is expressed on antigen-presenting cells to suppress T-cell activation (Li et al., 2017). The concurrent upregulation of TIGIT, PD-1, CTLA4, and BTLA points to the formation of a synergistic inhibitory network (Rotte et al., 2021; Sun et al., 2023). This synchronization strongly implies that *Bacillus* contributes to establishing an immunosuppressive milieu, effectively dampening anti-tumor immunity. This statistical correlation between microbial abundance and host dampening pathways was remarkably substantiated yet structurally refined by our high-resolution spatial FISH-IF tissue data (**Figure 6**). While bulk transcriptomic analysis indicated a generalized positive correlation between overall *Bacillus* load and checkpoint expressions across whole tissues, our *in situ* imaging unveiled a sophisticated, niche-specific spatial compartmentalization. We observed that within the high-load *Bacillus* niches, leukocytes exhibited a profound upregulation of CTLA-4 MFI, suggesting that the immediate microbial core is dominated by intensely suppressive elements. Conversely, PD-1 expression displayed an inverse spatial pattern, being significantly concentrated within the *Bacillus*-low peripheral regions. This spatial segregation strongly implies an immune exclusion phenotype, where high-load bacterial clusters drive immediate proximal CTLA-4 engagement while keeping PD-1+ effector leukocyte infiltrates marginated or excluded at the microbe-deficient boundaries, underscoring the active role of *Bacillus* in sculpting distinct microenvironmental niches to evade host surveillance.

Furthermore, *Bacillus* exhibited distinct correlations with specific immune cell subsets. While enrichments in plasma cells and memory B cells suggest an activation of humoral immunity, a significant negative correlation was observed with M1 macrophages. Given that the polarization from a pro-inflammatory M1 to an immunosuppressive M2 phenotype is a hallmark of tumor escape (Binnewies et al., 2018), the *Bacillus*-associated reduction in M1 infiltration likely impairs immune surveillance. Paradoxically, *Bacillus* also showed positive correlations with activated NK cells and Tfh cells. This dichotomy suggests that *Bacillus* exerts a pleiotropic, context-dependent effect on the TIME. Although different bacterial components or metabolites may trigger divergent immune pathways (Canzian et al., 2025; Garcia Castillo et al., 2025), the dominant association with checkpoint markers and reduced M1 macrophages indicates that the net effect favors immune evasion. These insights offer a novel perspective on resistance mechanisms, suggesting that targeting the intratumoral microbiome could enhance the efficacy of immune checkpoint inhibitors.

Several limitations of this study warrant consideration. First, the genus-level resolution of 16S rRNA sequencing precludes the identification of specific species or strains. Second, the observational nature of the study prevents establishing definitive causality between *Bacillus* and LUAD. Third, while our multiplexed FISH-IF provided robust spatial and morphological association profiles *in vivo*, active *in vitro* mechanistic interventions were not executed during this revision phase. This constraint was primarily dictated by the nature of our clinical cohorts, which consisted of historical flash-frozen and paraffin-preserved surgical specimens optimized for structural and nucleic acid preservation rather than specialized transport media required for viable microbial culturomics. Future prospective multi-center studies utilizing fresh-tissue microfluidics and germ-free experimental models will be essential to dissect the precise receptor-mediated cascades through which *Bacillus* components differentially modulate CTLA-4 and PD-1 spatial gradients. To address these, future multi-center studies should employ metagenomics, culturomics and experimental models to elucidate specific strains and mechanisms, while also integrating the virome and mycobiome for a more holistic ecological perspective.

## 5 Conclusion

In conclusion, this study provides the first evidence of the significant disparity between the intratumoral and bronchoalveolar lavage fluid microbiomes in LUAD, identifying *Bacillus* as a pivotal genus enriched within the tumor niche. Crucially, the abundance of *Bacillus* is closely associated with aggressive pathological features and an immunosuppressive microenvironment. These findings not only offer novel insights into the microbial ecology of lung cancer but also establish a theoretical foundation for developing microbiome-based diagnostic biomarkers and personalized immunotherapeutic strategies.

## Data Availability

The datasets presented in this study can be found in online repositories. The names of the repository/repositories and accession number(s) can be found in the article/**Supplementary Material**. The original clinical and sequencing contributions presented in the study are not readily available due to privacy and ethical restrictions. Requests to access the datasets should be directed to the corresponding author.
