# Supplementary material for: Paired comparison of tumor core and airway lumen (BALF) microbiomes in lung adenocarcinoma: deciphering specific Bacillus enrichment and immunomodulation
Source: Front Cell Infect Microbiol. 2026 Jul 6;16:1768287. doi: 10.3389/fcimb.2026.1768287 (PMC13381187; doi:10.3389/fcimb.2026.1768287)
Supplement: Supplementary file 1 [file Table1.docx]

|  | **P-values** | **FDR** | **TS** | **BALF** | **LDAscore** |
| --- | --- | --- | --- | --- | --- |
| **Enriched in BALF (Negative LDA)** | | | | | |
| *Bradyrhizobium* | 8.36E-25 | 7.90E-24 | 1298.0 | 105730 | -4.72 |
| *Not_Assigned* | 2.88E-12 | 5.44E-12 | 54904.0 | 144630 | -4.65 |
| *Ralstonia* | 3.22E-25 | 3.72E-24 | 724.8 | 87160 | -4.64 |
| *Staphylococcus* | 5.25E-21 | 2.10E-20 | 753.3 | 32567 | -4.20 |
| *Cutibacterium* | 1.08E-08 | 1.33E-08 | 9985.2 | 37254 | -4.13 |
| *Hyphomicrobium* | 4.30E-28 | 8.95E-27 | 0 | 28013 | -4.15 |
| *Mycobacterium* | 3.22E-23 | 1.76E-22 | 6845.5 | 34343 | -4.14 |
| *Paracoccus* | 6.83E-20 | 2.54E-19 | 1352.8 | 23077 | -4.04 |
| *Acinetobacter* | 2.59E-03 | 2.89E-03 | 43362.0 | 57554 | -3.85 |
| *Alcaligenes* | 3.04E-16 | 9.58E-16 | 1111.4 | 16985 | -3.90 |
| *Planococcus* | 4.30E-28 | 8.95E-27 | 0 | 13503 | -3.83 |
| *Rubellimicrobium's:Rubellimicrobium_roseum* | 4.30E-28 | 8.95E-27 | 0 | 13243 | -3.82 |
| ***Enriched in Tumor Tissue (Positive LDA)*** | | | | | |
| *Burkholderia_Caballeronia_Paraburkholderia* | 4.84E-11 | 7.86E-11 | 137670 | 66737 | 4.55 |
| *Bacillus* | 1.10E-23 | 7.64E-23 | 295280 | 15919 | 5.15 |
| *Mycoplasma* | 7.87E-26 | 1.02E-24 | 281510 | 725.65 | 5.15 |

**Table S3** Detailed statistics of differentially abundant taxa identified by LEfSe analysis.
