# Supplementary material for: Paired comparison of tumor core and airway lumen (BALF) microbiomes in lung adenocarcinoma: deciphering specific Bacillus enrichment and immunomodulation
Source: Front Cell Infect Microbiol. 2026 Jul 6;16:1768287. doi: 10.3389/fcimb.2026.1768287 (PMC13381187; doi:10.3389/fcimb.2026.1768287)
Supplement: Supplementary file 2 [file Table2.docx]

| **Functional Category** | **Mean Abundance**  **(BALF)** | **Mean Abundance**  **(Tumor Tissue)** | ***P*-value** |
| --- | --- | --- | --- |
| METABOLISM | 0.453 | 0.443 | <0.001 |
| CELLULAR PROCESSES AND SIGNALING | 0.241 | 0.237 | 0.048 |
| INFORMATION STORAGE AND PROCESSING | 0.188 | 0.204 | <0.001 |
| POORLY CHARACTERIZED | 0.118 | 0.116 | <0.001 |

**Table S4** Detailed statistical results of the functional enrichment analysis corresponding to Figure 4B.
