# Supplementary material for: Paired comparison of tumor core and airway lumen (BALF) microbiomes in lung adenocarcinoma: deciphering specific Bacillus enrichment and immunomodulation
Source: Front Cell Infect Microbiol. 2026 Jul 6;16:1768287. doi: 10.3389/fcimb.2026.1768287 (PMC13381187; doi:10.3389/fcimb.2026.1768287)
Supplement: Supplementary file 3 [file Table3.docx]

| **KEGG ID** | **Mean Abundance (BALF)** | **Mean Abundance (Tumor Tissue)** | **P-value** |
| --- | --- | --- | --- |
| K03088 | 0.0036 | 0.0043 | < 0.001 |
| K01990 | 0.0030 | 0.0040 | < 0.001 |
| K01992 | 0.0030 | 0.0039 | < 0.001 |
| K00059 | 0.0030 | 0.0032 | < 0.001 |
| K06147 | 0.0024 | 0.0028 | < 0.001 |
| K07090 | 0.0024 | 0.0023 | < 0.001 |
| K00626 | 0.0024 | 0.0017 | < 0.001 |
| K02014 | 0.0024 | 0.0017 | < 0.001 |
| K02035 | 0.0022 | 0.0019 | < 0.001 |
| K02015 | 0.0018 | 0.0026 | < 0.001 |

**Table S5** Detailed statistical results of the KEGG pathway analysis corresponding to Figure 4C.
