# Supplementary material for: Paired comparison of tumor core and airway lumen (BALF) microbiomes in lung adenocarcinoma: deciphering specific Bacillus enrichment and immunomodulation
Source: Front Cell Infect Microbiol. 2026 Jul 6;16:1768287. doi: 10.3389/fcimb.2026.1768287 (PMC13381187; doi:10.3389/fcimb.2026.1768287)
Supplement: Supplementary file 4 [file Table4.docx]

| Characteristics | All patients (N=77) |
| --- | --- |
| Age, years | 57.8 (83.0-31.0) |
| Sex |  |
| Male | 30 (39.0%) |
| Female | 47 (61.0%) |
| Tumor infiltration |  |
| AIS/MIA | 21 (27.3%) |
| IAC | 47 (61.0%) |
| NA | 9(11.7%) |
| Radiological features |  |
| GGN | 49 (63.6%) |
| Solid | 24(31.2%) |
| NA | 4(5.2%) |
| Clinical T Stage |  |
| T1 (a/b/c) | 47 (61.0%) |
| T2 | 5 (6.5%) |
| NA | 25(32.5%) |

**Table 1** Clinical characteristics of the patients
