# Supplementary material for: Paired comparison of tumor core and airway lumen (BALF) microbiomes in lung adenocarcinoma: deciphering specific Bacillus enrichment and immunomodulation
Source: Front Cell Infect Microbiol. 2026 Jul 6;16:1768287. doi: 10.3389/fcimb.2026.1768287 (PMC13381187; doi:10.3389/fcimb.2026.1768287)
Supplement: Supplementary file 5 [file Table5.docx]

| **Genus(Tumor Tissue)** | **Prevalence** |
| --- | --- |
| *Mycoplasma* | 1 |
| *Bacillus* | 1 |
| *Burkholderia_Caballeronia_Paraburkholderia* | 1 |
| *Achromobacter* | 0.797 |
| *Not_Assigned* | 0.623 |
| *Acinetobacter* | 0.623 |
| *Cutibacterium* | 0.261 |
| *Methylobacterium_Methylorubrum* | 0.246 |
| *Anoxybacillus* | 0.217 |

| **Genus(BALF)** | **Prevalence** |
| --- | --- |
| *Ralstonia* | 0.961 |
| *Burkholderia_Caballeronia_Paraburkholderia* | 0.947 |
| *Bradyrhizobium* | 0.934 |
| *Acinetobacter* | 0.789 |
| *Mycobacterium* | 0.671 |
| *Cutibacterium* | 0.566 |
| *Hyphomicrobium* | 0.513 |
| *Sphingomonas* | 0.487 |
| *Paracoccus* | 0.474 |
| *Achromobacter* | 0.474 |
| *Staphylococcus* | 0.421 |
| *Anoxybacillus* | 0.408 |
| *Pseudomonas* | 0.289 |
| *Kocuria* | 0.276 |
| *Alcaligenes* | 0.276 |
| *Brevundimonas* | 0.263 |
| *Bacillus* | 0.184 |

**Table S2** The prevalence of the top 20% most abundant genera in tumor tissue and BALF samples
